# Management of renal arteriovenous malformations: A pictorial review

**DOI:** 10.1007/s13244-014-0342-4

**Published:** 2014-07-05

**Authors:** Adam Hatzidakis, Michele Rossi, Charalampos Mamoulakis, Elias Kehagias, Gianluigi Orgera, Miltiadis Krokidis, Apostolos Karantanas

**Affiliations:** 1 Department of Medical Imaging, University Hospital of Heraklion, Medical School of Crete, 71110 Heraklion, Greece; 2 Department of Radiology, S. Andrea Hospital Sapienza Rome University, Rome, Italy; 3 Department of Urology, University Hospital of Heraklion, Heraklion, Crete Greece; 4 Department of Radiology, Cambridge University Hospitals NHS Trust, Cambridge, UK

**Keywords:** Renal arteriovenous malformation, Embolisation, Metallic coils, Onyx

## Abstract

**Background:**

Arteriovenous malformations (AVMs) are communications between an artery and a vein outside the capillary level. This pathologic communication may be either a fistula, a simple communication between a single artery and a dilated vein, or a more complex communication, a nidus of tortuous channels between one or more arteries/arterioles and one or more draining veins. The latter type of lesion is most frequently seen in the extremities; in the kidney they tend to appear more rarely. The most common clinical presentation of renal arteriovenous malformations (RAVMs) is haematuria. Percutaneous treatment with selective endovascular techniques offers a minimally invasive, nephron-sparing option in comparison to the more invasive surgical approaches. The purpose of this pictorial review is to highlight the general lines of management and to show the range of imaging findings of the percutaneous treatment of RAVMs.

**Methods:**

The imaging characteristics of a selection of cases of percutaneously managed congenital RAVMs are presented and the most common lines of approach are discussed.

**Conclusion:**

The imaging spectrum of diagnosis and percutaneous treatment of RAVMs is presented in order to aid interpretation and endovascular management.

***Teaching points*:**

• *Renal arteriovenous malformations are very rare lesions.*

• *Clinical expression is usually haematuria.*

• *Diagnosis is made with CT or MRI but the gold standard is digital subtraction angiography.*

• *Catheter-directed treatment with the use of coils or liquid embolics is minimally invasive, safe and effective.*

## Introduction

The abnormal communications between arteries and veins outside the capillary level are called arteriovenous malformations (AVMs) and result from a failed embryologic vascular development process. The dysplastic connection may be between a single arterial feeder and a draining vein that is usually enlarged, and this is the case of the arteriovenous fistulas or AVFs, or between a conglomerate of arterial branches and tortuous channels forming a nidus and one or more draining veins. Both types may be present in the renal vasculature in the form of either congenital renal arteriovenous fistulas (CRAVFs) or renal arteriovenous malformations (RAVMs).

Vascular and interventional radiologists should recognise such complex lesions and offer a definitive treatment under the elective or emergency setting whenever possible. The purpose of this pictorial review is to highlight the general lines of approach and to show the range of imaging findings of diagnosis and the percutaneous endovascular treatment of RAVMs.

## Aetiology

The abnormal communications between arteries and veins within the renal vasculature are acquired in approximately 75% of the cases and the result of an iatrogenic injury (most commonly biopsies), trauma or tumour [1–11]. Congenital RAVMs are significantly more rare, with reported incidence of 0.04% at autopsy. Approximately 200 cases have been reported to date [2, 11–15]. RAVMs and CRAVMs used to be classified as variceal and as cavernous malformations, respectively [4, 7, 9, 10]. However, since the introduction of the Mulliken and Glowacki classification for vascular malformations and the criteria set by the International Society for Study of Vascular Anomalies, these terms have been abandoned [10, 16]. RAVMs appear more frequently in women (3:1), more often involving the right kidney. They represent spontaneous failures of vascular development usually occurring between the 4th–10th week of embryonic life leading to dysplastic subepithelial vessel formation lacking elastic lamina, located in the calyceal or pelvic submucosa [17].

## Clinical presentation

The most common clinical presentation (75% of the cases) is macro- or microhaematuria [11, 18, 19]. Haematuria occurs because of dysplastic vessel rupture within the urinary collecting system and may become life threatening in many cases of major blood loss. The severity of haematuria is irrelevant to the size of the lesion; even small RAVMs may lead to severe blood loss if located near the pelvicalyceal system. If the blood loss is slow, clots may be formed that block the pelicalyceal system leading to urinary obstruction and flank pain. Hypertension may also be seen and may be related to altered flow dynamics within the renal artery. Congestive heart failure is usually seen in cases with relatively large congenital fistulas rather than RAVMs. In such cases the patients usually present with atrial fibrillation and symptoms of right heart failure such as raised arterial and blood jugular venous pressure, palpable liver edge, jaundice and lower limb oedema. However, symptoms of heart failure are usually delayed, and no clinical suspicion of the fistula is present. The incidence of atrial fibrillation is not clear.

## Imaging methods and findings

Assessment with ultrasound (US) using an abdominal 2–4-MHz convex probe may be the initial approach in some cases. RAVMs are hypoechoic lesions in the b-mode and flow-filled lesions in colour-Doppler imaging with high velocity (up to 60 cm/s) turbulent flow [20]. Turbulent flow with an arterial spectrum may also be detected within the inferior vena cava (IVC) in case a high-flow AVM is located in the right kidney. If the AVM is located in the left kidney, dilatation of the ipsilateral renal vein may be noticed.

Computed tomography (CT) is a very useful tool for the evaluation of RAVMs. A non-contrast-enhanced scan is usually performed to rule out the presence of urinary stones or calcifications. A biphasic scan is carried out in arterial and delayed venous phase (90–120 s) as per the haematuria protocol to investigate other potential causes of haematuria. The arterial phase images are expected to reveal the presence of tortuous small arteries with thin ramifications and in some cases the early filling of the IVC too. Small RAVMs may not be detectable with CT scans. Therefore, in case of persistent unexplained microhaematuria digital subtraction angiography (DSA; see below) is suggested to investigate the presence of a small RAVM.

**Fig. 1:**
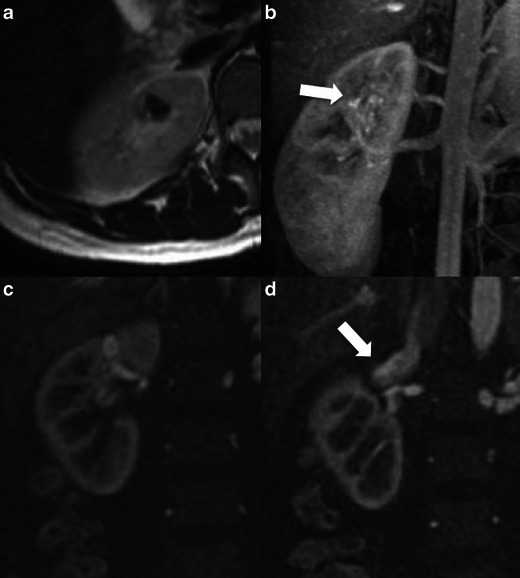
A 38-year-old woman was admitted to the emergency department because of acute right flank pain and severe macroscopic haematuria. Medical history revealed right-sided nephrolithiasis for which she had undergone three sessions of extracorporeal shock wave lithotripsy in the past. Laboratory investigation revealed moderate anaemia and normal renal function. Renal US ruled out hydronephrosis and neoplasia (not shown). A small (10-mm) residual stone fragment detected in a single calyx was not considered responsible for the haematuria. No ureteral stones were detected with a non-contrast CT scan. Diagnostic cystoscopy was within normal limits except for bleeding from the right ureteric orifice. Diagnostic right-sided semi-rigid ureteroscopy excluded any gross ureteral pathology.**a**-**d** MR imaging of the abdomen showed a small low-signal-intensity lesion in the upper lobe of the right kidney on T2-w TSE images (**a**). The lesion was not obvious on plain T1-w images. The 3D T1 gradient echo MR angiography showed early filling of a 1.5-cm lesion (**b**, *arrow*) located in the subcortical parenchyma (**c**) with early venous drainage directly to the inferior vena cava (**d**, *arrow*). Findings were more conspicuous on the original thin-slice-thickness images rather than the MIP reconstructions. These findings were consistent with RAVM

Magnetic resonance imaging (MRI) may also be used and in this case the RAVM appears as a flow-void area on T2-weighted (T2-w) images. Early filling of the IVC may be seen after i.v. contrast medium administration (Fig. 1). MRI might be indicated, instead of CT in young patients because of the lack of ionising radiation.

The gold standard for the diagnosis/characterisation of RAVMs is DSA, which offers the possibility to evaluate the lesion dynamically, delineate the feeder branches anatomically and plan the treatment. Retrograde puncture of the right common femoral artery is usually performed; a 4- or 5-F sheath is inserted followed by selective catheterisation of the renal artery. Automated pump injection (3–4 cc/s and 12 cc in total) of contrast medium is usually done via a 4- or a 5-F catheter located at the ostium of the renal artery and a 2-3 frames/s angiogram is performed. DSA will demonstrate the feeding arteries and super-selective catheterisation with the use of a microcatheter may follow, if necessary. Angiography of both kidneys is advisable, since rarely bilateral RAVMs may be present.

## Management

When haematuria is clinically manifested and the diagnosis of an RAVM has been established, urgent treatment planning is required. Especially if haematuria is life threatening, an emergency approach is necessary. Surgical options include total or partial nephrectomy [21]. However, surgical options are invasive, require several days of hospitalisation and are related to high morbidity because of the risk of complete loss of renal function and the risk of herniation of the lateral abdominal wall [22]. Therefore, percutaneous embolisation techniques have been developed for the management of such complex lesions [23–25]. Such procedures are performed under local anaesthesia in the majority of the cases.

Embolisation techniques aim at permanent occlusion of the multiple small channels between arteries and veins that form the nidus of the AVM and all the arterial feeders. The outcome of the procedure depends on the technique and the type of embolic material used. Usually several feeding arteries are involved at the segmental and interlobar level. Every feeding artery needs to be examined angiographically.

The embolic material used for the treatment of RAVMs may be Gelfoam, absolute alcohol, polyvinyl alcohol (PVA), coils and liquid embolic materials such as *n*-butyl 2-cyanoacrylate (NBCA) glue and ethylene vinyl alcohol copolymer (Onyx, Covidien, USA).

### Embolisation with gelfoam

Gelfoam or absorbable gelatin sponge has been used as the first choice in the past and has resulted in occlusion or diminishing of the AVM [26]. However, this approach is not permanent and haematuria may recur [10]. The Gelfoam embolisation technique is only mentioned for historical purposes and is not used in the current clinical practice of management of RAVMs.

### Embolisation with alcohol

Embolisation with absolute alcohol aims to ablate the lesion. The mechanism of alcohol ablation is not completely clear. It seems to be based on a combination of perivascular necrosis, endothelial damage, arterial spasm and sludging of erythrocytes leading to vessel occlusion [27]. The use of absolute alcohol was very popular in the past as it is very effective in occluding the nidus and acts against renin-dependent hypertension [28]. Migration to the pulmonary circulation did not raise concern, since the alcohol solution is usually very diluted.

In a publication of Mitchel et al. in 2006 [29], the role of alcohol was questioned in terms of complications considering that a case of cardiovascular collapse occurred. The pathophysiology of the collapse was not clear; however it was attributed mainly to direct toxicity of alcohol to the cardiac conduction system or to pulmonary artery spasm. After this case the authors monitored the pulmonary artery pressures during embolisaton with alcohol as a standard practice to determine whether alcohol induces pulmonary artery spasm and results in elevation of the pulmonary artery systolic pressure. After 92 procedures the authors reported a minor increase of the systolic blood pressure (2.3 mmHg) and of the systolic pulmonary artery pressure (1.0 mmHg), which was attributed to pain during the procedure. Therefore the cause of the severe but thankfully rare complications of alcohol was not clarified.

In order to be effective, alcohol must stagnate in contact with the endothelium at least for several seconds. Therefore, its use in high-flow malformations is not indicated, unless the in- and outflow is mechanically almost completely blocked. Reflux of alcohol in the arterial system can be prevented with the use of a balloon catheter (Berenstein Occlusion Balloon Catheter; Boston Scientific) that can be wedged into the arterial branch and inflated. Absolute alcohol needs to be slowly injected by hand at a rate of approximately 0.1–0.2 ml/s after a test injection at the same rate with the use of contrast material to confirm the absence of reflux. Each injection needs to be followed by the infusion of 1 ml of saline at the same rate. At the end of the injection the catheter needs to be slowly withdrawn and possible residual alcohol and debris need to be aspirated. Otherwise two long sheaths may be inserted from both the arterial and venous access and a balloon may be inflated from the venous end to avoid migration of alcohol in the venous system. More severe complications have been noticed when PVA particles are used. Nevertheless, embolisation with absolute alcohol is not easily controllable, not tolerated by all patients and may lead to complications. The main complications related to alcohol infusion are dyspnoea and headache. Symptoms are usually severe but last only for a couple of minutes*.*

Takebayashi et al. [10] reported their experience in 30 patients submitted to 34 embolisation procedures for controlling haematuria secondary to RAVMs over a 10-year period. Combinations of gelatin sponge, PVA particles and absolute alcohol were used. In 22 cases total and in 8 partial occlusion was achieved. After a mean follow-up of 6.2 years massive haematuria recurred in four patients; two were initially treated with gelatin sponges, one with the combination of alcohol and gelatin sponges and one with PVA. A significant percentage of renal parenchyma infarction (mean 15.7 %) occurred in nearly all patients. Non-target embolisation due to reflux of the embolic agents occurred in three patients. Alcohol injection caused transient dyspnoea and immediate headache in one patient. Infusion of alcohol or PVA into an RAVM with marked arteriovenous shunting may cause dyspnoea and headache, because less-diluted embolic material reaches the lungs and the brain. However, the small dose of alcohol that we use for ablation causes little tissue damage because dilution of alcohol below 50% eliminates its toxicity. In order to avoid this reaction, the alcohol should be injected slowly, at a rate of less than 0.2 ml/s, while the catheter is being placed selectively in the feeding vessel. Multiple small perfusion defects were detected with scintigraphy in the lung parenchyma in patients treated with PVA. Renin-dependent hypertension occurred in the same group of patients 6 months after embolisation. There was a slight increase of serum creatinine levels in 14 patients, but renal function remained within normal limits.

### Embolisation with coils

**Fig. 2:**
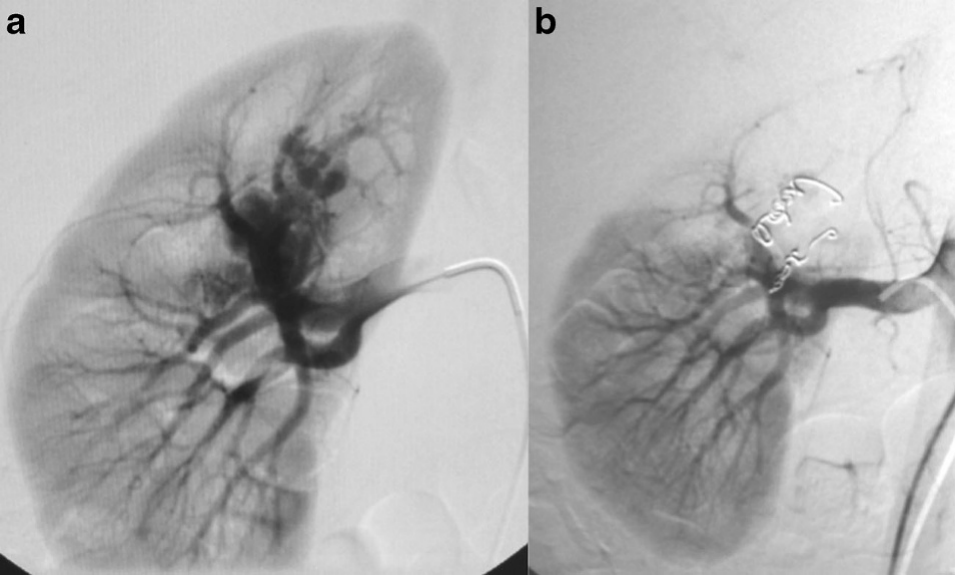
Same patient as in Fig. 1. **a** Emergency angiography with selective catheterisation of the right renal artery confirmed the presence of an upper renal pole AVM. **b** Two major feeding arterial branches supplied the AVM. Rapid venous filling through enlarged pool-like vascular structures was demonstrated. Both sites were successfully embolised with metallic coils of 3 × 2.6 (*n* = 3), 4 × 4.0 (*n* = 3) and 6 × 2.6 (*n* = 1) mm diameter (2D Helical-35, Boston Scientific, Cork, Ireland). The procedure was uneventful, haematuria subsided immediately and the patient recovered fully within the next few days

**Fig. 3:**
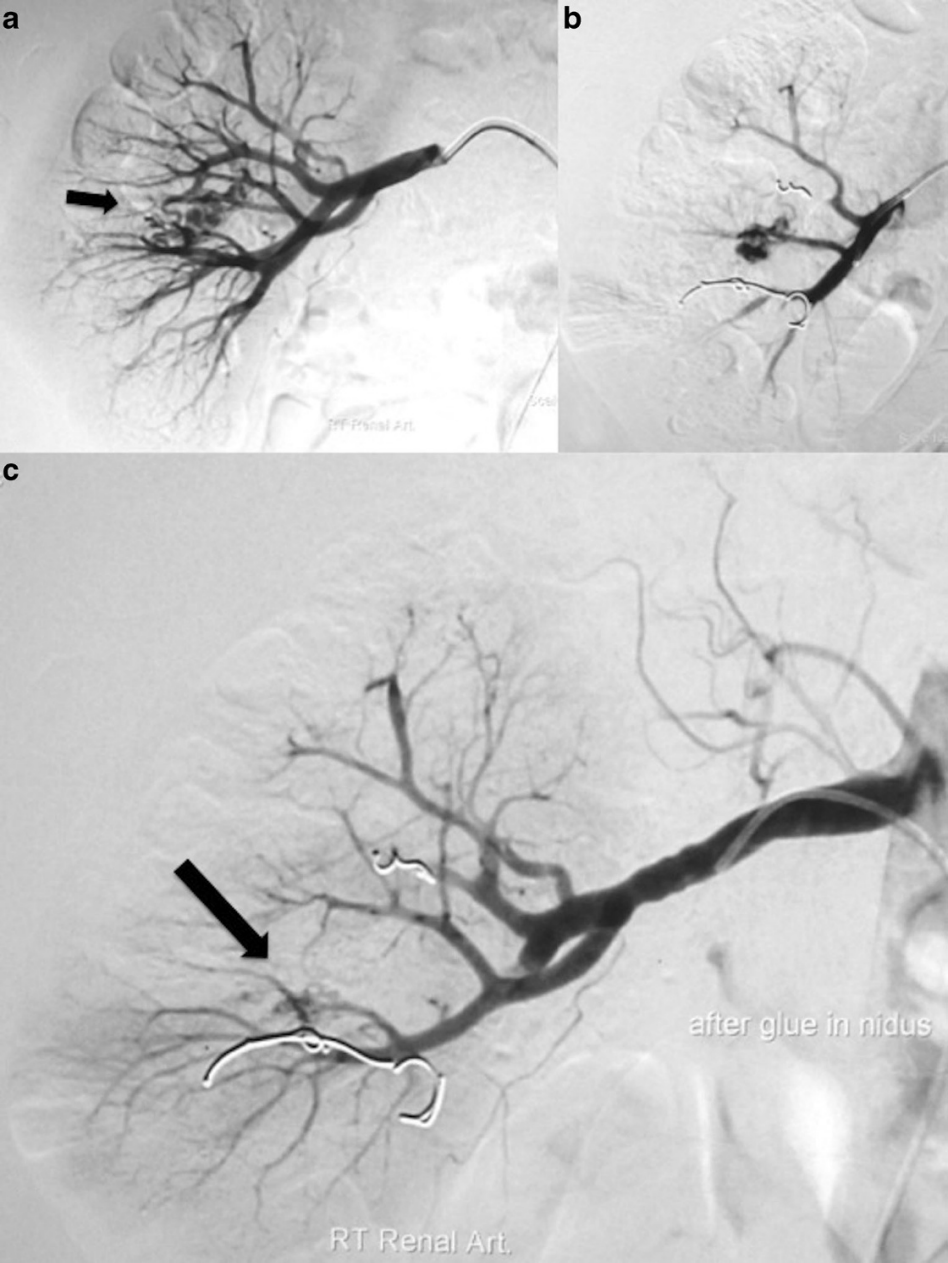
A 33-year-old man was admitted to the emergency department because of acute right flank pain and severe macroscopic haematuria and laboratory findings like the case described in Figs. 1 and 2. Contrast CT scan revealed a “bag-of-worms”-like mass in the middle portion of the right kidney (not shown). **a** Emergency angiography with selective catheterisation of the right renal artery confirmed an area of tortuous, coiled vascular channels with early filling of the renal vein within a few seconds after contrast injection in the centre of the kidney (arrow). These findings confirmed the RAVM diagnosis, which appeared to receive blood supply from three small arterial branches. **b** Two of the feeding branches were successfully embolised with metallic coils of 3 × 2.6 (*n* = 4) mm diameter (2D Helical-35, Boston Scientific, Cork, Ireland). **c** The middle feeding branch was closer to the nidus (arrow), and embolisation by injection of tissue NBCA glue (GlueBran2, GEM S.r.l., Viareggio, Italy) was chosen. The final angiogram showed a good post-embolisation result. The haematuria stopped and the patient recovered within the next days

The most commonly adopted embolic material for the treatment of RAVMs is metallic coils (or microcoils), which are mainly made from stainless steel or platinum [30–36] (Figs. 2 and 3). Coils, sized appropriately, offer a more controlled embolisation. The coils are deployed in the feeding branches of the lesion aiming to occlude the inflow. However, coils do not treat the nidus directly and success is not always guaranteed. Coils may also migrate and lead to pulmonary embolism, but this is very unusual in the treatment of RAVMs where the nidal vessels are small and most likely occurs in CRAVFs where aneurysmal dilatation of the veins is present. Protection balloons may be used in the arterial and venous system also in the case of coil embolisation as described for alcohol. Migration may be prevented with “caging” techniques as described by Durack et al. [37], where an OptEase IVC filter was used as a “cage” for coils. Yoon et al. [38] described a migration of coils in the colon post embolisation of a renal lesion due to erosion of the bowel wall from a local inflammatory reaction. However, judging from the number of coils (no angiographic picture is provided) the treated lesion was most likely a renal aneurysm rather than an RAVM.

### Embolisation with liquid embolics

The use of liquid embolics offers the possibility to penetrate deeper in the lesion and reach the nidus [23, 24, 39]. This is a tool that needs to be used in experienced hands because complications may occur in case of migration of the embolic material into the venous circulation.

Liquid embolic material may be either *n*-butyl 2-cyanoacrylate (NBCA) glue or ethylene vinyl alcohol copolymer (Onyx, Covidien Europe). NBCA glue can be mixed with lipiodol (usually 1:2) and after super selective nidus catheterisation slow injection follows. Once the nidus has been completely filled and the glue reaches the draining vein, injection needs to be stopped and the microcatheter is usually withdrawn within the carrying catheter (Fig. 3c). Before glue injection, catheter filling with 5% glucose is mandatory. The same fluid is used to clean the catheter after glue injection and during catheter retrieval in order to prevent adhesion of the catheter tip in the arterial vessel.

**Fig. 4:**
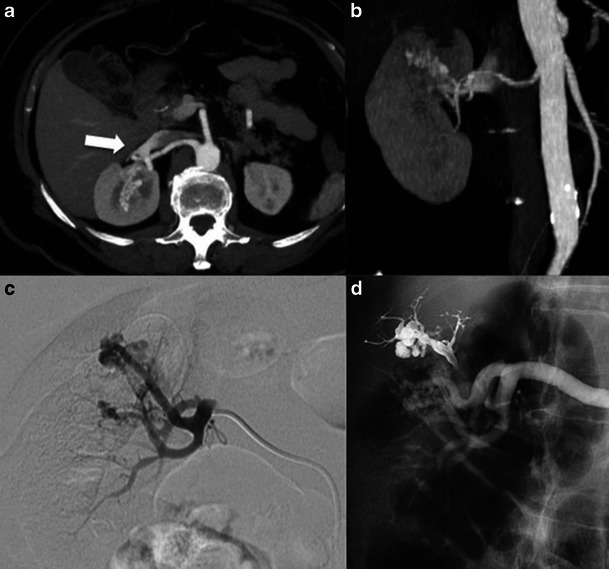
A 72-year-old woman was admitted to the emergency department because of right flank pain and macroscopic haematuria. Laboratory investigation revealed haemoglobin of 9.3 mg/dl. Abdominal US showed only multiple clots within the urinary bladder. **a**-**b** Contrast-enhanced CT scan revealed a conglomerate of vessels in the upper pole of the right kidney, visible in arterial phase with early filling of the IVC (arrow). The diagnosis of RAVM was made and the patient was submitted to a selective right renal angiogram using a hydrophilic 4-F Simmons 1 catheter. **c** Angiogram showed a tortuous nidus in the RAVM at the level of the upper renal pole with supply from two different superior branches without contrast blush, which would have suggested active bleeding. **d** Subsequent embolisation with injection of liquid embolic material (Onyx, eV3 Europe SAS, Paris, France); interval angiogram shows exclusion of the upper lesion. Angiogram of the lower lesion followed

Onyx is a copolymer of ethylene vinyl alcohol dissolved in dimethyl sulphoxide (DMSO). The composite is mixed with tantalum powder to allow visualisation under fluoroscopy. Embolisation procedures with the use of Onyx require catheters specifically designed for this liquid embolic material; otherwise there is a risk of damage of the inner layer of the catheter. In order to avoid premature precipitation of Onyx within the catheter, the dead space of the catheter is filled with DMSO. When the liquid embolic comes in contact with blood or interstitial fluids a cast is formed that hardens in a centripetal way. This offers the possibility of continues injection and remodelling, considering that the inner layer is still flexible. There is one case in the literature of Onyx use to treat RAVM [39]. The patient complained for persistent macroscopic haematuria and a small RAVM was revealed with DSA following a negative CT scan. A small amount (0.4 ml) of liquid embolic was used with a satisfactory result. A case of Onyx treatment is shown in Fig. 4.

Finally, the combination of metallic coil placement and glue injection is possible (Fig. 3). This approach can be used in cases where coil treatment seems insufficient, and thus additional glue injection can be performed. In case the appropriate coil size is not available or if the length of the feeding branch is quite short and there is a possibility of coil migration, then glue is a better option.

## Conclusion

RAVMs are rare lesions that may lead to severe haematuria. Diagnosis is based on the presence of tortuous vessels within the renal parenchyma in cross-sectional imaging, with early venous filling in the contrast-enhanced pictures. Treatment with transcatheter embolisation is based on the use of coils or liquid embolic agents as well as the combination of both.
